# In Vitro Bond Strength of Dentin Treated with Sodium Hypochlorite: Effects of Antioxidant Solutions

**DOI:** 10.3390/antiox13091116

**Published:** 2024-09-14

**Authors:** Guillermo Grazioli, Elisa de León Cáceres, Romina Tessore, Rafael Guerra Lund, Ana Josefina Monjarás-Ávila, Monika Lukomska-Szymanska, Louis Hardan, Rim Bourgi, Carlos Enrique Cuevas-Suárez

**Affiliations:** 1 Department of Dental Materials, School of Dentistry, Universidad de la República, Av. General Las Heras 1925, Montevideo 11300, Uruguay; ggrazioli@odon.edu.uy (G.G.); elisadeleon5@gmail.com (E.d.L.C.); rominatessore@gmail.com (R.T.); 2 Department of Restorative Dentistry, School of Dentistry, Federal University of Pelotas, Pelotas 96010-610, Brazil; rglund@ufpel.edu.br; 3 Dental Materials Laboratory, Academic Area of Dentistry, Autonomous University of Hidalgo State, San Agustín Tlaxiaca 42160, Mexico; ana_monjaras@uaeh.edu.mx; 4 Department of General Dentistry, Medical University of Lodz, 92-213 Lodz, Poland; monika.lukomska-szymanska@umed.lodz.pl; 5 Department of Restorative Dentistry, School of Dentistry, Saint-Joseph University, Beirut 1107 2180, Lebanon; louis.hardan@usj.edu.lb; 6 Department of Digital Dentistry, AI and Evolving Technologies, Saint-Joseph University, Beirut 1107 2180, Lebanon; 7 Department of Biomaterials and Bioengineering, INSERM UMR_S 1121, University of Strasbourg, 67000 Strasbourg, France

**Keywords:** antioxidants, bond strength, dentin, root canal treatment, dental adhesives, meta-analysis, resin-based materials, sodium hypochlorite, systematic review

## Abstract

This systematic review aims to evaluate whether the application of antioxidant solutions can enhance the bond strength of resin-based materials to sodium hypochlorite (NaOCl)-treated dentin. This study follows the PICOT strategy: population (sodium hypochlorite-treated dentin), intervention (application of antioxidants), control (distilled water), outcome (bond strength), and type of studies (in vitro studies). The systematic review and meta-analysis were conducted following PRISMA guidelines. Electronic databases were searched for in vitro studies evaluating the effects of antioxidants on bond strength to sodium hypochlorite-treated dentin. Two independent reviewers screened articles, extracted data, and assessed risk of bias. Meta-analyses were performed using a random-effects model to compare standardized mean differences in bond strength between antioxidant pretreatment and control groups. Inclusion criteria consisted of in vitro studies that examined the bond strength of resin-based materials to NaOCl-treated dentin with antioxidant application, while exclusion criteria included studies with incomplete data, those not using a control group, or those that did not directly measure bond strength. From 3041 initial records, 29 studies were included in the qualitative analysis and 25 in the meta-analysis. Ascorbic acid, sodium ascorbate, grape seed extract, green tea, and rosmarinic acid significantly improved bond strength to sodium hypochlorite-treated dentin (*p* < 0.05). The effectiveness of grape seed extract varied with adhesive system type. Hesperidin, p-toluene sulfonic acid, and sodium thiosulfate did not significantly improve bond strength. Most studies had a high risk of bias. This suggests that the conclusions drawn from these studies should be interpreted with caution, and further research with more robust methodologies may be needed to confirm the findings. In conclusion, this systematic review implies that certain antioxidants can improve bond strength to sodium hypochlorite-treated dentin, with efficacy depending on the specific agent and adhesive system used. Further standardized studies are needed to optimize protocols and confirm these findings.

## 1. Introduction

Sodium hypochlorite (NaOCl) is commonly used during root canal treatment as an irrigant due to its ability to dissolve organic tissues and disinfect the root canal system [1]. However, this irrigation solution has been shown to negatively affect the bond strength between resin-based materials and dentin [2]. NaOCl causes oxidation of dentin collagen fibrils, interfering with resin infiltration and subsequent resin tag formation [3]. This results in the reduced bond strength of resin-based composite materials, resin cements, and adhesive systems to NaOCl-treated dentin [4].

Various antioxidants have been proposed as potential pretreatments to reverse the negative effects of NaOCl on dentin bonding. Antioxidants are substances that can inhibit oxidation by neutralizing free radicals and reactive oxygen species. Studies have shown that antioxidants like ascorbic acid, proanthocyanidins, and rosmarinic acid, among others, can restore compromised bond strength after NaOCl treatments [5]. However, other antioxidants like sodium thiosulfate have demonstrated limited efficacy [6].

Antioxidants such as ascorbic acid and sodium ascorbate act by donating electrons to reactive oxygen species generated by NaOCl, effectively reducing these oxidants and preventing them from causing further harm to the dentin matrix [7]. Additionally, antioxidants like grape seed extract and green tea contain polyphenolic compounds that scavenge free radicals [8]. These polyphenols are capable of neutralizing free radicals before they can interact with and degrade dentin components. Furthermore, certain antioxidants can form stable complexes with oxidative species, thereby reducing their reactivity and mitigating their adverse effects on dentin. By interrupting the oxidative chain reactions initiated by NaOCl, these antioxidants help preserve the integrity of the dentin and maintain the bond strength between resin-based materials and NaOCl-treated dentin [9].

Despite promising results with some antioxidants, there is heterogeneity within the existing literature evaluating their effects on bond strength. Variability exists in terms of the specific antioxidant used, its pH and concentration, application protocol, dentin substrate, bonding system, testing method, and storage conditions [10]. A previous review on this topic had a narrow focus only on total-etch adhesives [11]. No recent systematic reviews have thoroughly summarized the current evidence on this topic across different adhesive strategies.

For all of the aforementioned, the objective of this systematic review is to analyze whether the application of antioxidant solutions can improve the bond strength of resin-based materials to sodium hypochlorite-treated dentin. The hypothesis of this review would be that the use of antioxidant solutions could improve the compromised bond strength of resin-based materials to sodium hypochlorite-treated dentin.

## 2. Materials and Methods

### 2.1. Protocol and Registration

This systematic review was registered on the Open Science Framework (OSF; https://osf.io (accessed on 5 September 2024)) with the identifier DOI 10.17605/OSF.IO/UKEM9. This review adhered to the recommendations outlined in the Preferred Reporting Items for Systematic Reviews and Meta-Analysis (PRISMA) statement [12].

### 2.2. Information Sources and Search Strategy

The search strategy (Table 1) was initially developed for the MEDLINE database using specific keywords for each component of the PICOT strategy: population (sodium hypochlorite-treated dentin), intervention (application of antioxidants), control (distilled water), outcome (bond strength), and type of studies (in vitro studies). The MEDLINE search strategy was modified for other electronic databases, including Scielo, Web of Science, Scopus, and Embase. Additionally, the first 100 results from Google Scholar were also consulted. The search was conducted on 25 June 2023.

### 2.3. Selection Process and Data Collection Process

After conducting the search strategy, an online software program (Rayyan (https://www.rayyan.ai/), Qatar Computing Research Institute, HBKU, Doha, Qatar) was utilized to store files from all databases and detect duplicates. The same software program was used to assess the title and abstract of the articles. This phase involved two independent reviewers who checked whether the articles met the following inclusion criteria: (1) in vitro studies investigating the effect of the application of antioxidants solution prior to the adhesion strategy on the bond strength of resin-based materials to sodium hypochlorite-treated dentin; (2) evaluated the bond strength of adhesive systems to the aforementioned substrate using either resin-based composite materials or resin-based cement as antagonists; (3) included a control group; (4) reported mean and standard deviation (SD) data in MPa for shear, microshear, micro-tensile, and tensile bond tests; and (5) available in English, Spanish, or Portuguese. Case series, case reports, pilot studies, and reviews were excluded. Additionally, studies involving caries-affected dentin were excluded since it was addressed in subsequent analyses.

Each eligible article was assigned a study identification code by combining the last name of the first author with the publication year. The same two reviewers reviewed and categorized data, including the antioxidant used, the conditions of the dentin substrate, the application protocol for the antioxidant, the bond strength test, and the storing time of the samples before the bond strength test. In the event of any disagreement between reviewers, a third expert opinion was sought to provide additional insights and facilitate the resolution process.

### 2.4. Quality Assessment

Two reviewers independently assessed the risk of bias in the included manuscripts using the parameters of previous systematic reviews [13,14]. The risk of bias assessment focused on seven key parameters. The tool used evaluated if the authors reported the following parameters or not: specimen randomization, single operator, operator blinded, standardized specimens, sound teeth, sample size calculation, and control group. The studies were evaluated for the randomization of specimens to ensure unbiased allocation to different groups. The consistency of procedures performed by a single operator was examined to reduce inter-operator variability. The assessment also included verification of whether the operator was blinded to group allocation during the experiment, which is an important measure to minimize potential bias. Standardized protocols for specimen preparation and treatment were reviewed to ensure consistency across experiments. The use of sound, non-carious teeth in the experiments was another critical factor considered. The adequacy of sample size calculations was scrutinized to confirm sufficient statistical power. Finally, the inclusion of an appropriate control group was evaluated as a fundamental aspect of robust experimental methodology. Collectively, these parameters provided a comprehensive framework for assessing the methodological quality and potential biases in the studies.

If the authors reported the parameter, then the article received a YES in such parameter, otherwise the article received a NO. The risk of bias was classified as low, medium, or high as a function of the total of yeses obtained according to the following scale: 1–3 as high, 4–5 as medium, and 6–7 as low.

### 2.5. Statistical Analysis

A meta-analysis was conducted using Review Manager version 5.3.5 (The Cochrane Collaboration, Copenhagen, Denmark). A random-effect model was employed, and estimates were obtained by comparing the standardized mean difference between bond strength values in groups where an antioxidant solution pretreatment was used before the bonding procedures versus the control group. Separate analyses were performed for each antioxidant found. Subgroup analyses were performed according to the type of adhesive used.

## 3. Results

**Figure 1:**
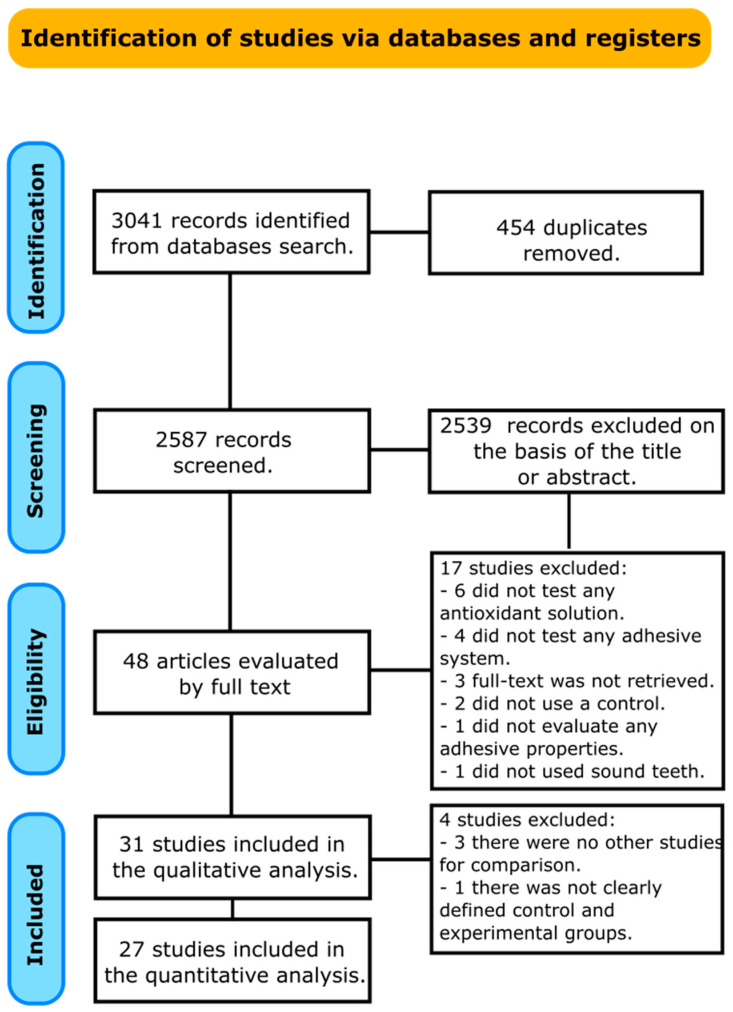
PRISMA flow diagram of study selection process.

A total of 3041 papers were obtained from all databases. After removing duplicate entries, 2587 documents were reviewed by examining their titles and abstracts. Following this initial screening, 48 studies remained for a thorough examination of the full text. Among these, 17 were excluded for the following reasons: in 6, an antioxidant solution was not used [15,16,17,18,19,20]; in 4, an adhesive system was not tested [21,22,23,24] l3 studies lacked accessible full-text manuscripts [25,26,27]; in 2, a control group was not identified [28,29]; in 1, the bond strength was not tested [30]; and in 1, the bond strength was tested on caries-affected dentin [31]. A total of 31 articles were included in the qualitative analysis. However, four studies were excluded from the quantitative analysis. Three of these studies were removed because they lacked comparable data or similar study designs, making it impossible to perform a meaningful statistical comparison [32,33,34]. The fourth study was excluded because the control and experimental groups were not clearly defined, leading to potential inconsistencies in the data analysis [35]. Finally, 27 articles remained for the meta-analysis. Figure 1 illustrates the selection process according to the PRISMA statement.

Table 2 shows the main characteristics of the studies included in the qualitative analysis. The antioxidants retrieved by this systematic review that were used in order to restore the bond strength of resin-based materials to sodium hypochlorite-treated dentin were as follows: grape seed extract/proanthocyanidin, tannic acid, green tea, *N-acetyl cysteine*, sodium thiosulfate, ascorbic acid, sodium ascorbate, *p-toluene* sulfonic acid, citric acid, hesperidin, riboflavin, propolis, rosmarinic acid, phytic acid, rosmarinic acid, and epigallocatechin. The studies evaluated the effect of these substances on the bond strength of total-etch, self-etch, and self-adhesive resin cements using shear, tensile, micro-shear, micro-tensile, and push-out bond strength tests. Most of the studies evaluated only the immediate bond strength.

**Figure 2:**
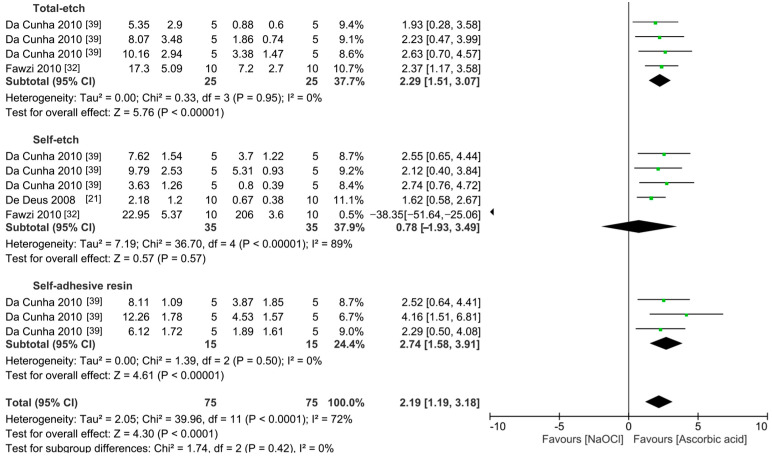
Forest plot showing the effect of the application of ascorbic acid as pretreatment on the bond strength to sodium hypochlorite-treated dentin.

**Figure 3:**
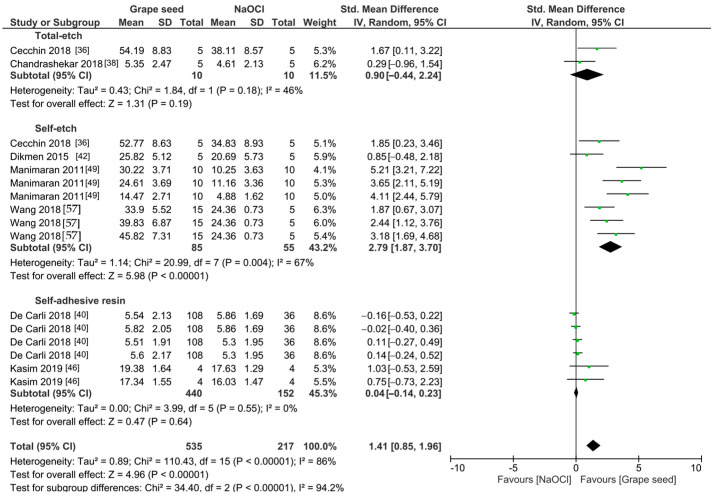
Forest plot showing the effect of the application of grape seed as pretreatment on the bond strength to sodium hypochlorite-treated dentin.

**Figure 4:**
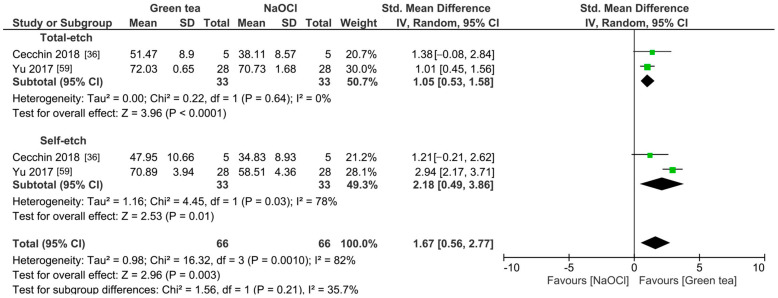
Forest plot showing the effect of the application of green tea as pretreatment on the bond strength to sodium hypochlorite-treated dentin.

**Figure 5:**
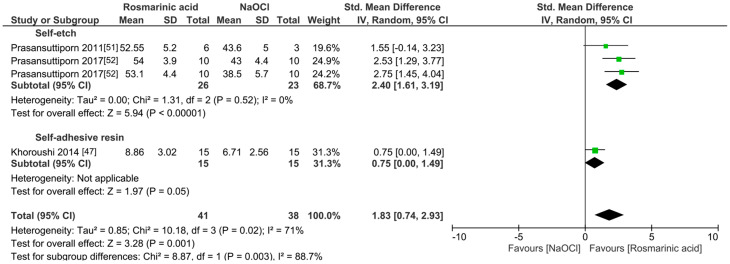
Forest plot showing the effect of the application of rosmarinic acid as pretreatment on the bond strength to sodium hypochlorite-treated dentin.

**Figure 6:**
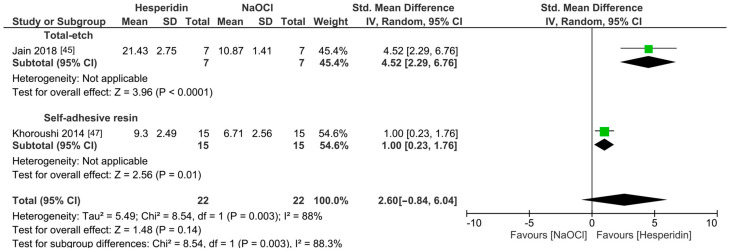
Forest plot showing the effect of the application of hesperidin as pretreatment on the bond strength to sodium hypochlorite-treated dentin.

**Figure 7:**
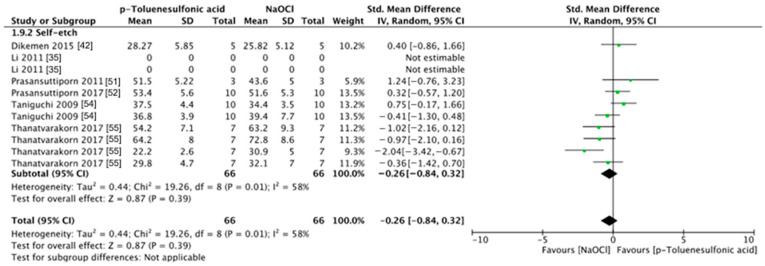
Forest plot showing the effect of the application of *p-toluene* sulfonic acid as pretreatment on the bond strength to sodium hypochlorite-treated dentin.

**Figure 8:**
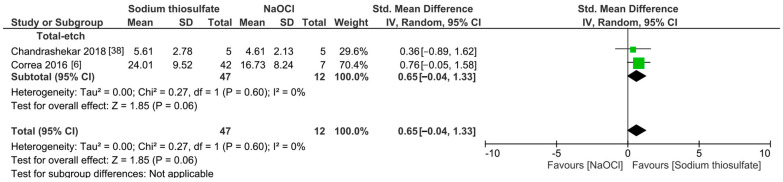
Forest plot showing the effect of the application of sodium thiosulfate as pretreatment on the bond strength to sodium hypochlorite-treated dentin.

**Figure 9:**
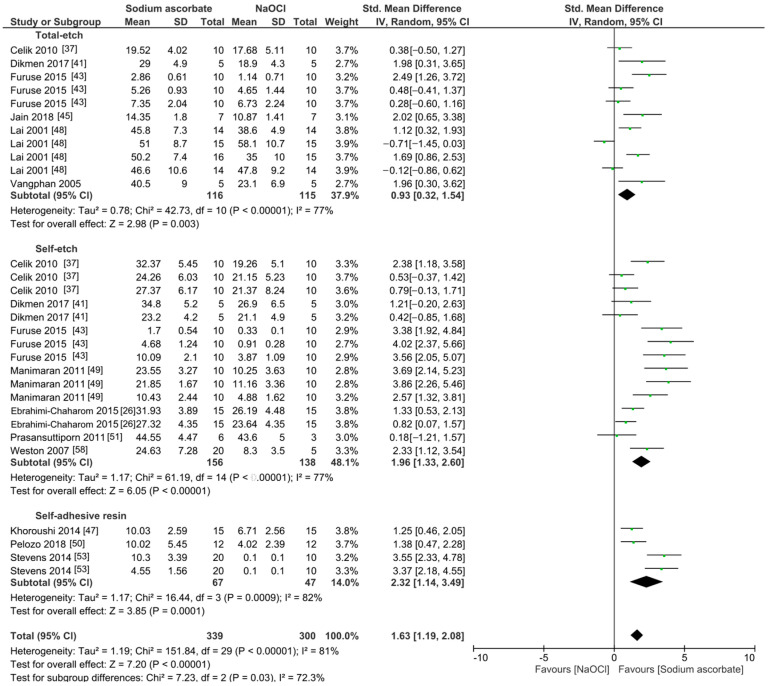
Forest plot showing the effect of the application of sodium ascorbate as pretreatment on the bond strength to sodium hypochlorite-treated dentin.

The meta-analyses were conducted according to the antioxidant evaluated. Separate meta-analyses for each antioxidant to evaluate their individual effects on bond strength to sodium hypochlorite-treated dentin were conducted. Additionally, subgroup analyses were conducted according to the type of adhesive used to assess how different adhesive systems influence the efficacy of the antioxidants. These analyses were aimed at identifying specific patterns and understanding how various factors impact the overall findings. Figure 2, Figure 3, Figure 4, Figure 5, Figure 6, Figure 7, Figure 8 and Figure 9 show these results.

The effect of the application of ascorbic acid as pretreatment on the bond strength to sodium hypochlorite-treated dentin is showed in Figure 2. According to the results, regardless of the adhesive system used, the use of this antioxidant can improve the bond strength to sodium hypochlorite-treated dentin (*p* < 0.001).

The improvement in the bond strength values is observed too for the use of grape seed as a pretreatment (*p* < 0.0001). However, this effect is only observed when self-etch adhesives are used (*p* < 0.001), while for total-etch (*p* = 0.19) and self-adhesive resins (*p* = 0.64), this effect was not observed (Figure 3). When green tea was used as a pretreatment prior to the application of total-etch or self-etch adhesives systems (Figure 4), the bond strength was also improved (*p* = 0.003). This effect was also observed when rosmarinic acid (Figure 5) was used (*p* = 0.001).

Overall, the bond strength to sodium hypochlorite-treated dentin was not improved when hesperidin was used as a pretreatment (Figure 6, *p* = 0.14). Despite this, it is worth mentioning that the meta-analysis of this group included only one article per adhesive material. Also, the use of *p-toluene* sulfonic acid did not improve the bond strength (Figure 7, *p* = 0.39). Likewise, sodium thiosulfate did not improve the bond strength of a resin-based material to sodium hypochlorite-treated dentin (Figure 8, *p* = 0.06).

Finally, when sodium ascorbate was used as a pretreatment, the bond strength was improved (Figure 9, *p* < 0.001).

A substantial heterogeneity was observed across the studies included in this review, as indicated by the I^2^ statistics. To address this, we performed subgroup analyses to examine how different study characteristics influenced the results. Despite these analyses, the high level of heterogeneity remains a limitation, affecting the interpretability and generalizability of the results.

The risk of bias analysis is presented in Table 3. According to the domains analyzed, most of the articles were catalogued as having a high risk of bias. The parameters where most of the studies failed were single operator, operator blinded, and control group.

## 4. Discussion

This systematic review analyzed the effect of various antioxidant pretreatments on bond strength of resin materials to NaOCl-treated dentin. The results of the meta-analysis showed that the application of certain antioxidants prior to bonding procedures can effectively improve compromised bond strength caused by NaOCl irrigation. However, the efficacy differed based on the specific antioxidant used.

To provide a deeper understanding of the varying efficacy of antioxidants, the chemical properties and interactions of specific antioxidants with dentin were explored. For example, grape seed extract, rich in polyphenols, exhibits strong antioxidant properties due to its ability to scavenge free radicals and stabilize oxidative species [60]. Its effectiveness may be influenced by its polyphenolic content, which interacts differently with dentin compared to other antioxidants. In contrast, sodium ascorbate, a derivative of ascorbic acid, functions primarily through electron donation to reduce oxidative species. Its role in mitigating oxidative damage in dentin may be less pronounced compared to grape seed extract due to differences in its mechanism of action and stability in the presence of dentin components [61]. Understanding these differences in chemical properties and their implications on dentin bonding can provide insights into the selection of antioxidants for clinical applications and highlight the need for further research to optimize their use based on their specific interactions with dentin substrates.

Ascorbic acid, sodium ascorbate, grape seed extract, green tea, and rosmarinic acid were able to significantly enhance bond strength to NaOCl-treated dentin. The improvement seen with ascorbic acid and sodium ascorbate is likely due to their ability to scavenge free radicals and reduce oxidized dentin collagen to reversible hydrophilic aldehydes and ketones [61]. Catechins in green tea and proanthocyanidins in grape seed extract provide similar antioxidative protection [62]. Rosmarinic acid contains phenolic compounds that may quench the reactive oxygen species introduced by NaOCl [51].

In contrast, antioxidants like hesperidin, *p-toluene* sulfonic acid, and sodium thiosulfate showed no significant difference versus control. The lack of efficacy with sodium thiosulfate was surprising given prior evidence of its antioxidant capacity [6]. However, the limited number of studies evaluating these particular antioxidants precluded definitive conclusions. Interestingly, grape seed extract only improved bond strength for self-etch adhesives, whereas ascorbic acid worked for both etch-and-rinse and self-etch systems. The exact mechanisms behind this adhesive-specific effects remain unclear and warrant further investigation.

Overall, the most effective antioxidant pretreatments appear to share common attributes of low pH and the ability to reduce oxidized compounds back to original states. However, the optimal protocol for antioxidant usage to maximize bond strength is still uncertain. Heterogeneity existed across the included studies in terms of antioxidant concentration, pH, application time, adhesive type, and testing methods.

Several studies have explored different approaches to improving the bond strength of resin composite to sodium hypochlorite-treated dentin. Ishizuka et al. [63] investigated the effect of varying application times of NaClO on dentin bonding, providing valuable insight into how timing can influence adhesion. Di Francescantonio et al. [64] examined how different adhesives affect the formation of the acid-base resistant zone and bond strength after NaClO treatment. In another study, Wang et al. [57] focused on the recovery effects of proanthocyanidin, emphasizing the importance of antioxidant concentration in reversing the oxidative damage caused by NaClO on dentin.

In the context of resin-based materials bonding to sodium hypochlorite-treated dentin and the longevity of resin-based composite materials, various risk factors have been identified. These factors can be categorized into patient-level, dentist-related, and tooth/restoration-related factors [65,66]. Patient-level factors include caries risk, parafunctional habits, number of check-ups per year, and socioeconomic status. Dentist-related factors involve different operators and their experience levels. Tooth/restoration-related factors encompass endodontic treatment, tooth type, and the number of restored surfaces.

Of particular importance for restoration durability is the presence of endodontic treatment [67], which has been shown to increase the risk of restoration failure by more than two times. Other risk factors at the tooth and dentition levels have been less frequently studied. For instance, teeth with one or no adjacent teeth were found to have twice the risk of fractures compared to teeth with two neighboring teeth [68]. In contrast, the presence of proximal contacts was shown to be a protective factor for restoration survival. It is plausible that proximal contacts help distribute occlusal forces [69], while teeth without two adjacent teeth could be second molars or part of a mutilated dentition with other related risk factors.

Moreover, additional variables can be considered as indirect factors related to general oral health conditions, lifestyle, and motivational issues. For example, bleeding on probing has been associated with a negative influence on the survival of cervical restorations, possibly indicating poorer levels of oral hygiene. Similarly, the presence of a removable denture may negatively impact restoration survival [70].

Regarding the dentin substrate, it is crucial to acknowledge that dentin’s composition is not fixed but rather dynamic and influenced by various factors. The relative position of dentin within the tooth, the age of the dentin, and the presence or absence of disease all play a significant role in shaping its composition [71] Consequently, comprehending the structural changes in dentin resulting from age, disease, or trauma is essential for advancing improved dentin adhesives. However, research efforts in this domain have encountered challenges.

Moreover, a significant portion of our knowledge regarding dentin bonding has been derived from in vitro bond strength studies conducted on intact, flat polished, and healthy dentin samples. While these results are highly valuable for comparing different commercial bonding systems, it is important to note that sound and healthy dentin is not the most common substrate encountered in clinical practice. In reality, clinicians often face the challenge of bonding adhesives to caries-affected (c-a) dentin or abraded-sclerotic dentin. Caries-affected dentin refers to the dentin that has been affected by decay or caries, which can lead to structural changes and compromised bonding characteristics. Abraded-sclerotic dentin, on the other hand, is dentin that has undergone wear and subsequent sclerosis, resulting in a different surface composition and topography compared to normal dentin [72].

Bonding to these altered dentin substrates poses unique difficulties due to their distinct properties. Therefore, it is crucial for dental researchers and clinicians to explore and understand the bonding behavior of adhesives to caries-affected and abraded-sclerotic dentin, as this knowledge is more reflective of real-world clinical scenarios and can aid in the development of more effective bonding strategies for such challenging situations. Overall, understanding and addressing these risk factors are crucial for optimizing the bond strength of resin-based materials to sodium hypochlorite-treated dentin and enhancing the long-term success of resin-based composite materials.

Finally, we conducted a critical evaluation of the methodologies employed in the articles included in this review. Several key methodological differences were identified that could influence the outcomes. Firstly, the application protocols for antioxidants varied significantly among the studies. These variations included differences in application time, ranging from a few seconds to several minutes, the methods of application such as brushing or soaking of the dentin, and the number of applications. Such discrepancies in application protocols can substantially impact the bond strength results, making it challenging to directly compare outcomes across different studies. Secondly, the concentrations of antioxidants used in the studies differed widely. Some studies employed low concentrations, while others used higher concentrations. The efficacy of the antioxidants and the resulting bond strength can be influenced by these variations in concentration. Therefore, it is crucial to consider the concentration of antioxidants as a variable when interpreting and comparing the findings.

This variability in study methodologies has significant implications for the overall conclusions. These differences contribute to high heterogeneity, affecting the comparability of results across studies. Consequently, this variability makes it challenging to draw consistent conclusions and underscores the need for cautious interpretation of the findings. Future research should focus on standardizing methodologies to enhance comparability and improve the reliability of evidence. Standardizing experimental protocols, including antioxidant application concentrations, durations, and techniques, is essential. This standardization will reduce variability between studies and allow for more accurate comparisons of results.

Additionally, this review was limited by the high risk of bias among the included studies. Many lacked methodological rigor related to randomization, blinding, standardized specimens, and sample size calculation. There was also an absence of long-term bond strength data. Further high-quality in vitro studies are needed to corroborate these results prior to clinical recommendation.

To enhance the clinical relevance of our findings, it is crucial to provide practical recommendations for clinicians regarding the selection and use of antioxidants in endodontic procedures. The results of our study highlight the significant potential of antioxidants, such as grape seed extract and sodium ascorbate, in improving bond strength. The application of antioxidants must follow standardized protocols to ensure consistency and effectiveness. Moreover, the integration of antioxidants into endodontic procedures should be considered with regard to their compatibility with adhesive systems. Clinicians should evaluate how the chosen antioxidants interact with specific adhesive materials to prevent any adverse effects on bonding performance.

## 5. Conclusions

The findings of this review suggest that antioxidants such as ascorbic acid, sodium ascorbate, grape seed extract, green tea, and rosmarinic acid can improve the bond strength of resin-based materials to sodium hypochlorite-treated dentin. Clinicians may consider using these antioxidants to enhance bonding effectiveness. However, due to the high risk of bias in the included studies, the results should be interpreted with caution. Future research with more rigorous methodologies is necessary to validate these findings and establish standardized protocols for clinical use.

To strengthen the evidence base and facilitate the development of robust, evidence-based clinical guidelines, it is imperative that future research adheres to more rigorous methodologies. Specifically, the adoption of standardized protocols across studies is crucial. Standardization will not only enhance the consistency and comparability of results but also support the formulation of reliable clinical recommendations. Emphasizing methodological rigor and uniformity in future research will ultimately contribute to more accurate and actionable insights into the use of antioxidants in clinical practice.

## Figures and Tables

**Table 1:** Search strategy used for the MEDLINE database.

Number	Search Terms
# 1	NaOCl OR NaOCl-treated dentin OR Sodium Hypochlorite OR NaOCl-Induced OR sodium hypochlorite-treated dentine OR smear layer-deproteinizing OR Oxidized Etched Dentin OR Deproteinized Dentin OR Root Dentin Deproteinization
# 2	antioxidant OR reducing agent OR Sodium Ascorbate OR Proanthocyanidin OR Grape seed extract OR ascorbic acid
# 3	Bond OR Bonding OR Dental bonding OR Bonding efficacy OR bond strength OR Bonding performance OR bonding effectiveness OR Bond performance OR adhesive properties OR microtensile strength OR Micro-tensile strength OR bonding properties OR Microtensile bond strength OR shear bond strength OR microshear bond strength OR performance
# 4	# 1 AND # 2 AND # 3

**Table 2:** Main characteristics of the studies included in the qualitative analysis.

Study	Antioxidants Used	NaOCl Treatment	Adhesive System Evaluated	Antioxidant Protocol Application	Bond Strength Test	Aging
Cecchin 2018 [36]	Grape seed extract Tannic acid Green tea *N-acetyl cysteine*	5.25% for 30 min	Etch-and-rinse: Single Bond Plus (3M ESPE) Universal: Scotchbond Universal (3M ESPE)	5 mL of 10% solution for 5 min	Microtensile	Distilled water for 24 h at 37 °C
Celik 2010 [37]	Sodium thiosulfate	5.25% NaOCl for 30 min	Etch-and-rinse: Scotchbond Multi-Purpose 3M	5 mL 0.5% to 5% for 1, 5, or 10 min	Microtensile	Distilled water for 24 h at 37 °C
Chandrashekhar 2018 [38]	Sodium thiosulfate Proanthocyanidin	3% NaOCl for 30 min	Etch-and-rinse: One Coat SL (Coltene)	5% sodium thiosulfate or proanthocyanidin solution for 10 min	Microtensile	Distilled water for 24 h at 37 °C
Correa 2016 [6]	Sodium thiosulfate	5.25% NaOCl for 30 min	Etch-and-rinse: Scotchbond Multi-Purpose (3M ESPE)	0.5% or 5% sodium thiosulfate used for 1, 5, or 10 min	Microtensile	Distilled water for 24 h at 37 °C
Da Cunha 2010 [39]	Ascorbic acid	5.25% NaOCl for 10 min	Self-adhesive resin cement: Rely X U100 (3M ESPE) Etch-and-rinse: Adper Single Bond 2 (3M ESPE) Self-etch: Clearfil SE Bond (Kuraray)	10% ascorbic acid for 10 min	Push-out	Distilled water for 24 h at 37 °C
De Carli 2018 [40]	Grape seed extract	0.9% sodium chloride solution	Self-adhesive resin cement: RelyX U200 (3M ESPE)	6.5% or 10% grape seed extract for 30, 60, or 120 s	Push-out	Distilled water for 24 h and 12 months at 37 °C
De Deus 2008 [21]	Citric acid	1.25% NaOCl	Self-etch primer: Epiphany self-etch primer (Resilon Research LLC)	5 mL of BioPure MTAD (citric acid)	Micropush-out	Distilled water for 7 days at 37 °C
Dikmen 2017 [41]	Sodium ascorbate	5.25% NaOCl for 30 s	Etch-and-rinse: Adper Single Bond 2 (3M ESPE) Self-etch: Clearfil SE Bond (Kuraray) Self-etch: Xeno III (Dentsply)	10% sodium ascorbate for 10 min	Microtensile	Distilled water for 24 h at 37 °C
Dikmen 2015 [42]	Grape seed extract *p-toluene* sulfonic acid	5.25% NaOCl for 30 s	Universal: Single Bond Universal (3M ESPE)	5% grape seed extract *or* *p-toluene* sulfonic acid solution for 10 min	Microtensile	Distilled water for 24 h at 37 °C
Ebrahimi-Chaharom [26]	Sodium ascorbate	5.25% NaOCl for 10 minutes	Self-etch: Clearfil S3 Bond (Kuraray) and Adper Easy One (3M ESPE)	10% sodium ascorbate for 10 minutes.	Shear	Distilled water for 24 h at 37°C and then 500 rounds thermocycling at 5/55 °C
Fawzi 2010 [32]	Citric acid	5.25% NaOCl for 10 min	Self-etch: Clearfil S3 Bond (Kuraray) Etch-and-rinse: Adper Single Bond 2 (3M ESPE)	Citric acid for 5 min	Micro shear	Distilled water for 24 h at 37 °C
Furuse 2015 [43]	Sodium ascorbate	5.25% NaOCl for 10 min	Etch-and-rinse: Scotchbond Multi- Purpose Plus (3M ESPE) Self-etch: Xeno III (Dentsply)	10% sodium ascorbate during 10 min	Push-out	Distilled water for 24 h at 37 °C
Gönülol 2015 [44]	Sodium ascorbate	5.25% NaOCl for 10 min	Self-etch: Clearfil SE Bond (Kuraray)	10% sodium ascorbate solution for 10 min	Microtensile	Distilled water for 24 h at 37 °C
Jain 2018 [45]	Sodium ascorabte Hesperidin Riboflavin	3% NaOCl	Self-adhesive resin cement: Rely X Arc (3M ESPE)	10% sodium ascorbate or hesperidin for 4 min, 1% riboflavin for 4 min	Push-out	Not mentioned
Kalyoncuoğlu 2015 [33]	Propolis	5.25% NaOCl for 10 min	Self-etch: Clearfil. SE Bond (Kuraray)	20% propolis solution for 5 min	Micro shear	Distilled water for 1 week at 37 °C
Kasim 2019 [46]	Proanthocyanidin	5.25% NaOCl for 1 min	Self-adhesive resin cement: U-Cem (Vericom)	1 ml of Proanthocyanidin for 1 min	Push-out	Not mentioned
Khoroushi 2014 [47]	Rosmarinic acid Hesperidin Sodium ascorbate	5.25% NaOCl for 1 min	Self-adhesive resin cement: Bifix SE (Voco)	10% of each solution for 2 min	Push-out	Distilled water for 24 h at 37 °C
Lai 2001 [48]	Sodium ascorbate	5.25% NaOCl for 1 to 10 min	Etch-and-rinse: Single bond (3M ESPE) Etch-and-rinse: Excite (Ivoclar Vivadent)	10% sodium ascorbate for 1 to 10 min	Microtensile	Distilled water for 24 h at 37 °C
Li 2011 [35]	Sodium toluene sulfinic acid	10% NaOCl for 1 min	Self-etch: Super-Bond C&B (Sun Medical)	Sodium toluene sulfinic acid for 10 s	Microtensile	Distilled water for 24 h at 37 °C
Manimaran 2011 [49]	Proanthocyanidin agent/grape seed extract Sodium ascorbate	5.25% NaOCl for 15 to 20 min	Self-etch: Adper Bond (3M ESPE)	10% proanthocyanidin or sodium ascorbate for 10 min	Microtensile	Distilled water for 24 h at 37 °C
Nassar 2020 [34]	Phytic acid	5.25% NaOCl for 5 min	Universal: Scotchbond Universal (3M ESPE)	1% phytic acid for 1 min	Microtensile	Distilled water for 24 h at 37 °C
Pelozo 2018 [50]	Sodium ascorbate	1% NaOCl	Self-adhesive resin cement: RelyX U200 (3M ESPE)	10% sodium ascorbate for 10 min	Push-out	Distilled water for 24 h at 37 °C
Prasansuttiporn 2011 [51]	Sodium ascorbate Rosmarinic acid *p-toluene* sulfinic acid salt	6% NaOCl for 30 s	Self-etch: Clearfil Protect Bond (Kuraray).	10% antioxidant for 5 or 10 s	Microtensile	Distilled water for 24 h at 37 °C
Prasansuttiporn 2017 [52]	Rosmarinic acid *p-toluene* sulfinic acid salt	6% NaOCl for 30 s	Self-etch: Clearfil SE bond (Kuraray)	10% antioxidant solution for 5 s	Microtensile	Distilled water for 24 h at 37 °C
Stevens 2014 [53]	Sodium ascorbate	6% NaOCl	Etch-and-rinse: Excite (Ivoclar Vivadent) Self-Adhesive Resin Cement: Multilink (Ivoclar Vivadent) Self-etch: Clearfil DC Bond (Kuraray) Self-adhesive resin cement: SpeedCEM (Ivoclar Vivadent) Self-adhesive resin cement: Clearfil SA Cement (Kuraray)	10% sodium ascorbate for 5 s	Shear	Not mentioned
Taniguchi 2009 [54]	*p-toluene* sulfinic acid salt	6%NaOCl for 15 or 30 s	Self-etch: Clearfil DC Bond (Kuraray)	*p-toluene* sulfinic acid salt for 30 s	Microtensile	Distilled water for 24 h at 37 °C
Thanatvarakorn 2017 [55]	*p-toluen*e sulfinic acid salt	hypochlorous acid solution for 15 s	Self-etch: Clearfil Bond SE One (Kuraray) Universal: Scotchbond Universal (3M ESPE) Self-etch: BeautiBond Multi (Shofu) Self-etch: Bond Force (Tokuyama).	*p-toluene* sulfinic acid salt for 5 s	Microtensile	Distilled water for 24 h at 37 °C
Vangphan 2005 [56]	Sodium ascorbate	5.25% NaOCl for 10 min	Etch-and-rinse: Single bond (3M-ESPE)	10% sodium ascorbate for 10 min	Microtensile	Distilled water for 24 h at 37 °C
Wang 2018 [57]	Proanthocyanidin	5.25% NaOCl for 20 min	Self-etch: Clearfil SE Bond (Kuraray)	5, 10 or 15% Proanthocyanidin for 1, 5, or 10 min	Microtensile	Distilled water for 24 h at 37 °C
Weston 2007 [58]	Sodium ascorbate	5.25% NaOCl for 1 min	Self-etch: Clearfil SE Bond (Kuraray) Etch-and-rinse: Single bond 2 (3M-ESPE)	10% sodium ascorbate for 1, 3, and 10 min	tensile	Distilled water for 24 h at 37 °C
Yu 2017 [59]	Epigallocatechin	5.25% NaOCl for 1 min	Self-adhesive resin cement: U-Cem (Vericom)	10 mL of Epigallocatechin	Push-out	Distilled water for 24 h at 37 °C

**Table 3:** Qualitative synthesis (risk of bias assessment).

Study	Specimen Randomization	Single Operator	Operator Blinded	Standardized Specimens	Sound Teeth	Sample Size Calculation	Control Groups	Risk of Bias
Cecchin 2018 [36]	YES	NO	NO	YES	YES	YES	YES	Medium
Celik 2010 [37]	YES	NO	NO	NO	YES	NO	NO	High
Chandrashekhar 2018 [38]	YES	NO	NO	YES	NO	NO	YES	High
Correa 2016 [6]	YES	NO	NO	YES	NO	YES	YES	Medium
Da Cunha 2010 [39]	YES	YES	NO	YES	NO	YES	NO	Medium
De Carli 2018 [40]	YES	NO	NO	YES	YES	NO	YES	Medium
De Deus 2008 [21]	YES	NO	NO	YES	YES	NO	YES	Medium
Dikmen 2017 [41]	NO	NO	NO	YES	YES	YES	NO	High
Dikmen 2015 [42]	YES	NO	YES	YES	YES	YES	NO	Medium
Ebrahimi-Chaharom [26]	YES	NO	NO	YES	YES	NO	YES	Medium
Fawzi 2010 [32]	YES	NO	NO	YES	YES	YES	NO	Medium
Furuse 2015 [43]	NO	NO	NO	YES	NO	YES	NO	High
Gönülol 2015 [44]	YES	NO	NO	YES	YES	NO	YES	Medium
Jain 2018 [45]	NO	NO	NO	YES	YES	YES	NO	High
Kalyoncuoğlu 2015 [33]	YES	NO	NO	YES	NO	YES	NO	High
Kasim 2019 [46]	YES	NO	NO	YES	NO	YES	NO	High
Khoroushi 2014 [47]	YES	NO	NO	NO	YES	NO	YES	High
Lai 2001 [48]	NO	NO	NO	NO	NO	YES	YES	High
Li 2011 [35]	NO	NO	NO	YES	NO	NO	NO	High
Manimaran 2011 [49]	YES	NO	NO	YES	YES	NO	YES	Medium
Nassar 2020 [34]	NO	NO	NO	YES	YES	NO	NO	High
Pelozo 2018 [50]	NO	NO	NO	YES	NO	YES	NO	High
Prasansuttiporn 2011 [51]	NO	NO	NO	YES	YES	YES	NO	High
Prasansuttiporn 2017 [52]	YES	NO	NO	YES	YES	YES	NO	Medium
Stevens 2014 [53]	NO	NO	NO	NO	YES	NO	NO	High
Taniguchi 2009 [54]	NO	NO	NO	YES	YES	YES	NO	High
Thanatvarakorn 2017 [55]	YES	NO	NO	YES	YES	NO	YES	Medium
Vangphan 2005 [56]	NO	NO	NO	YES	YES	YES	NO	High
Wang 2018 [57]	YES	NO	NO	YES	YES	YES	NO	Medium
Weston 2007 [58]	YES	NO	NO	YES	YES	NO	NO	High
Yu 2017 [59]	YES	NO	NO	YES	NO	YES	NO	High

## Data Availability

The data presented in this study are available upon reasonable request from the author (G.G.).
